# Supplementary material for: The genome sequence of the Oriental Meadow Brown, Cercyonis lupina (Lepidoptera: Nymphalidae)
Source: Wellcome Open Res. 2026 Feb 22;11:141. [Version 1] doi: 10.12688/wellcomeopenres.25788.1 (PMC13036442; doi:10.12688/wellcomeopenres.25788.1)
Supplement: Peer review history

# Reviewer report: Reviewer response for version 1

**DOI:** 10.21956/wellcomeopenres.28389.r150600

**Published:** 2026-03-30

**Authors:** Frederique Hilliou

**Affiliations:** 1 Universite Cote d'Azur, Provence-Alpes-Côte d'Azur, France

The manuscript presents the genome of the Oriental Meadow Brown (Cercyonis lupina), a species of butterfly in the family Nymphalidae, subfamily Satyrinae. *C. lupina* is found in north Africa, southern Europe, Anatolia and Arabia, and in some part of Central Asia where it feeds on Poaceae. Two different lineages are found, based on DNA bar-coding, grouping north Africa and Iberian Peninsula versus the rest of the population. Divergence has been inferred ca. 3 Mya. The sample used here for full genome sequencing is an adult female collected in Catalonia.

 The haplotype 1 was curated at chromosome level (508.65Mb; 30 chromosomes including W and Z, 116 scaffolds, 172 contigs, and a 15.22kb mitochondrion) and the haplotype 2 at scaffold level (467.75Mb; 48 scaffolds, 109 contigs). BUSCO analysis using the lepidoptera_odb10 reference set identified 98.4% of this gene set in haplotype 1.

 The assembly metric for haplotype 1 is 7.C.Q64 according to the Earth BioGenome Project Report on Assembly Standards September 2024.

 Position of Merian elements is given across chromosomes of the haplotype 1.

 What is the % of heterozygoty between the 2 haplotypes?

 What was the method used for this genome annotation?

 The mitochondrial genome is available (OZ243821.1) however I was not able to find its annotation.

  The acquisition of this *C lupina* genome should help in the taxonomic classification of this species. 

Are sufficient details of methods and materials provided to allow replication by others?

Yes

Is the rationale for creating the dataset(s) clearly described?

Yes

Are the datasets clearly presented in a useable and accessible format?

Yes

Are the protocols appropriate and is the work technically sound?

Yes

Reviewer Expertise:

NA

I confirm that I have read this submission and believe that I have an appropriate level of expertise to confirm that it is of an acceptable scientific standard.

# Reviewer report: Reviewer response for version 1

**DOI:** 10.21956/wellcomeopenres.28389.r150596

**Published:** 2026-03-27

**Authors:** Liang Lü

**Affiliations:** 1 Hebei Normal University, Shijiazhuang, Hebei, China

The authors present a valuable de novo genome assembly for the Oriental Meadow Brown butterfly (Cercyonis lupina), utilizing modern long-read sequencing and Hi-C technologies. While generating high-quality genomic resources for non-model Lepidoptera is a commendable contribution to comparative genomics, the manuscript currently reads more like a technical data report than a comprehensive scientific article. Assuming the journal's scope accommodates purely descriptive data notes, I have two specific methodological concerns that still need to be addressed:

 1. Treatment of Heterozygosity: Lepidopteran genomes are known for their high levels of heterozygosity. Although the authors describe the assembly of a primary pseudo-haplotype, the manuscript lacks a sufficient discussion or characterization of the alternate haplotigs. But it can be left to other data-miners.

 2. Depth of Annotation: The current functional annotation is insufficient. Simply mapping ancestral linkage groups (Merian elements) does not constitute a thorough genomic annotation. The authors state that the genome "will be annotated" using available RNA-Seq data via Ensembl. This suggests a reliance on purely automated pipelines. For a resource of this type, it is highly recommended to include manual curation of ecologically significant gene families, which are vital for understanding butterfly biology.

Are sufficient details of methods and materials provided to allow replication by others?

Yes

Is the rationale for creating the dataset(s) clearly described?

Yes

Are the datasets clearly presented in a useable and accessible format?

Yes

Are the protocols appropriate and is the work technically sound?

Yes

Reviewer Expertise:

phylogenomics, entomology

I confirm that I have read this submission and believe that I have an appropriate level of expertise to confirm that it is of an acceptable scientific standard.
